# Librarians as methodological peer reviewers for systematic reviews: results of an online survey

**DOI:** 10.1186/s41073-019-0083-5

**Published:** 2019-11-27

**Authors:** Holly K. Grossetta Nardini, Janene Batten, Melissa C. Funaro, Rolando Garcia-Milian, Kate Nyhan, Judy M. Spak, Lei Wang, Janis G. Glover

**Affiliations:** Harvey Cushing/John Hay Whitney Medical Library, Yale University, 333 Cedar Street, New Haven, CT 06520-8014 USA

**Keywords:** Editorial policy, Evidence synthesis, Information specialists, Librarians, Meta-analysis, Methodological standards, Peer review, PRESS, Reporting standards, Systematic reviews

## Abstract

**Background:**

Developing a comprehensive, reproducible literature search is the basis for a high-quality systematic review (SR). Librarians and information professionals, as expert searchers, can improve the quality of systematic review searches, methodology, and reporting. Likewise, journal editors and authors often seek to improve the quality of published SRs and other evidence syntheses through peer review. Health sciences librarians contribute to systematic review production but little is known about their involvement in peer reviewing SR manuscripts.

**Methods:**

This survey aimed to assess how frequently librarians are asked to peer review systematic review manuscripts and to determine characteristics associated with those invited to review. The survey was distributed to a purposive sample through three health sciences information professional listservs.

**Results:**

There were 291 complete survey responses. Results indicated that 22% (*n* = 63) of respondents had been asked by journal editors to peer review systematic review or meta-analysis manuscripts. Of the 78% (*n* = 228) of respondents who had not already been asked, 54% (*n* = 122) would peer review, and 41% (*n* = 93) might peer review. Only 4% (*n* = 9) would not review a manuscript. Respondents had peer reviewed manuscripts for 38 unique journals and believed they were asked because of their professional expertise. Of respondents who had declined to peer review (32%, *n* = 20), the most common explanation was “not enough time” (60%, *n* = 12) followed by “lack of expertise” (50%, *n* = 10).

The vast majority of respondents (95%, *n* = 40) had “rejected or recommended a revision of a manuscript| after peer review. They based their decision on the “search methodology” (57%, *n* = 36), “search write-up” (46%, *n* = 29), or “entire article” (54%, *n* = 34). Those who selected “other” (37%, *n* = 23) listed a variety of reasons for rejection, including problems or errors in the PRISMA flow diagram; tables of included, excluded, and ongoing studies; data extraction; reporting; and pooling methods.

**Conclusions:**

Despite being experts in conducting literature searches and supporting SR teams through the review process, few librarians have been asked to review SR manuscripts, or even just search strategies; yet many are willing to provide this service. Editors should involve experienced librarians with peer review and we suggest some strategies to consider.

## Background

Systematic reviews (SR) summarize and evaluate primary studies on a research topic to establish evidence about the efficacy of an intervention [1]. When a systematic review is done well, it is considered to be evidence of the highest level on which to base health care decisions [2]. Systematic reviews (SR) and other types of evidence syntheses, for example meta-analyses, scoping reviews, integrative reviews, rapid reviews, and clinical guidelines, use rigorous protocols and guidelines to gather and synthesize all literature relevant to a research or clinical question [1, 3]. The methodology requires a systematic, transparent, reproducible, and comprehensive search to locate all studies, published and unpublished, about a topic [4, 5]. Conversely, narrative literature reviews do not require the same level of rigor in the literature search, nor the double screening of results to determine if the information found meets pre-established inclusion criteria. A literature review does not typically include formal quality assessment or risk of bias assessment and is not considered evidence-based.

The number of published systematic reviews is increasing dramatically; one study reports an increase of 2,700% between 1991 and 2014 [6]. Unfortunately, many of these SRs are conflicted, overlapping, and poorly reported [6]. Some research shows possible improvement in reporting quality, particularly in Cochrane SRs [7]. Methodological rigor, such as the quality of the search strategies, directly affects the quality of systematic reviews.

Identifying a comprehensive body of potentially relevant studies from the literature is a critically important initial step in an evidence synthesis and, if done poorly, can compromise the entire review [8, 9]. “Data” in a systematic review are the set of studies resulting from the comprehensive literature search, which is analogous to the findings and data from a primary research study or specific results or data from an experiment [9]. Proper construction, validation, and reporting of search strategies to retrieve these data are fundamental to the quality and reproducibility of systematic reviews and meta-analyses [4, 5, 7, 9–13]. Librarians, information specialists, and informationists are experts in searching for information, and systematic review quality improves when the systematic search methodology is designed and performed by a librarian [10, 14, 15]. Involvement of a librarian in the process of developing and executing a comprehensive search is increasingly evident in the literature and strongly encouraged by organizations such as the Cochrane Collaboration; the National Academies of Science, Engineering, and Medicine (previously Institute of Medicine—IOM); and the Campbell Collaboration [1, 4, 9]. Increasingly, because of expertise in searching and methodological advice, librarians are sought after as partners and co-authors of systematic reviews [16] and research shows that the quality of SRs is higher if librarians are included as co-authors [12]. At the authors’ institution, research teams undertaking systematic reviews with significant librarian involvement are required to include the librarian as a co-author [17–21].

Biomedical journal editors have expressed interest in improving the quality of published evidence syntheses, both in design and in reporting. Several journals now include specific systematic review instructions for authors or have appointed section editors especially for systematic reviews and other review types [14, 22, 23]. Some journals put submissions through statistical review as a matter of policy or encourage editors to pursue statistical review of certain sections by methodological specialists [24, 25]. Some journals require that authors use specific reporting standards for systematic reviews [26, 27]. Multiple standards exist for the design and reporting of systematic reviews as well as other evidence syntheses, chief among them IOM, Cochrane Handbooks, Methodological Expectations for Cochrane Intervention Reviews (MECIR), Meta-analyses Of Observational Studies in Epidemiology (MOOSE), and Preferred Reporting Items for Systematic Reviews and Meta-Analyses (PRISMA) [1, 4, 28–30]. Evaluation tools have also been developed for critical appraisal of systematic reviews (AMSTAR) and of SR search strategies (PRESS) [31, 32]. The EQUATOR Network provides a robust list of reporting guidelines for many study types [33]. If these standards are recommended by editorial policies and used by authors, then peer reviewers of systematic review and meta-analysis manuscripts should also use them as they conduct a review of the design, execution, and reporting of a systematic review manuscript [34]. Reviewers are also best positioned to effectively peer review if they have both subject expertise and experience with the study design of the manuscript they are evaluating. Librarians and information specialists with search expertise are well qualified to peer review the methodology and reported search strategies of SR manuscripts.

Librarians' roles in the systematic review process are broadening [35, 36]. However, the extent of librarians’ involvement as journal manuscript peer reviewers has not been investigated. This study sought to answer that question.

## Methods

A survey was developed to capture experiences of medical librarians with regard to the peer review process of SR manuscript submissions to journals. The 16-question survey was developed in Qualtrics, pilot tested with a group of medical librarians, and reviewed by a methodological expert. The survey questions included demographic information about type of professional setting, years as a librarian, and depth of involvement in systematic review teams. Questions were also asked about respondents’ experience with systematic reviews and/or peer reviewing. Survey logic presented different questions to different respondents (e.g., only those answering yes to a specific question would be asked questions related to that answer. The Yale University Human Subjects Committee ruled that this survey was exempt from human subjects protection (IRB #2000022848). The survey and a CHERRIES-compliant reporting checklist are Additional file 1: Tables S5 and S6 [37].

The survey was distributed to a purposive sample [38]. On March 15, 2018, the survey was emailed through three listservs known to be used by biomedical information professionals who do systematic reviews (AASHL-all, medlib-l, expertsearching) and a reminder notification was sent to the same listservs on March 29, 2018. To get wide distribution, recipients were encouraged to invite others to respond, a variation on a snowball sample. There were no financial incentives for participation. The survey closed on April 6, 2018. We were unable to calculate a response rate because respondents self-selected to complete the survey and were asked to invite additional respondents. Additionally, it is difficult to calculate the response rate because there is an unknown but potentially significant overlap between the three listserv subscriber groups. The survey data was extracted and analyzed using Microsoft Excel and R (version 3.5.3; The R Project for Statistical Computing). To analyze the association between variables, we used Fisher’s exact test. Preliminary results were reported at the Medical Library Association Meeting in May 2018 [39].

## Results

A total of 291 respondents completed the survey. The number of respondents per question ranged from 20 to 291. All results are presented in Additional file 1: Table S1.

The median number of years that respondents had been librarians was 11.5 (IQR 6–20). Most respondents worked in either an academic medical library (*n* = 169, 66%) or a teaching hospital (*n* = 37, 14%, Q12-13). More than a third of respondents (*n* = 95, 37%) had participated in over 11 systematic review teams or created and conducted searches for SRs. About one quarter (*n* = 61, 24%) had participated in 5–10 systematic reviews, another third (*n* = 79, 31%) had participated in 1–4 systematic reviews, and the remainder (*n* = 24, 9%) had never participated in or created and conducted searches for systematic reviews (Q14).

Respondents gained their expertise through three main methods: classes/webinars (*n* = 220, 85%), self-training (*n* = 145, 56%), or in-house training (*n* = 121, 47%, Q15). Most respondents had not been asked by a journal editor to peer review (*n* = 228, 78%). Of the respondents who had not been asked to peer review, most indicated that they would (*n* = 122, 54%) or might (*n* = 93, 41%) peer review a manuscript if asked. Only nine respondents said they would not peer review a manuscript if asked (*n* = 9, 4%, Q2, Q16).

For librarians who had been asked by a journal editor to peer review (*n* = 63, 22%), respondents listed 38 unique journal titles. They included *PLOS ONE* with five mentions and *JAMIA*: *a Scholarly Journal of Informatics in Health and Biomedicine*, *JBI Database of Systematic Reviews*, *Journal of the Medical Library Association*, and *Systematic Reviews* all with three mentions each (Additional file 1: Table S2). Most of the respondents (*n* = 31, 70%) knew why they were asked to peer review. The most frequent reasons given were their professional expertise, referral by a colleague, and expertise in the topic area (Q3-4).

The median number of systematic reviews or meta-analysis manuscripts that any one respondent peer reviewed was four (IQR 1–5), with one librarian having peer reviewed 40 manuscripts (Q5).

The survey included two questions that asked respondents to identify if they used any standards for evaluating the methods section or the search strategy. Over half of the respondents (*n* = 37, 59%) stated that they used standards to evaluate manuscripts’ methods sections. PRISMA was the most frequently mentioned methods standard (*n* = 32, 86%), followed by Cochrane (*n* = 10, 27%) and MECIR (*n* = 4, 11%, Additional file 1: Table S3). The respondents also identified the standards or checklists they utilized for evaluating search strategies (*n* = 36, 57%). Most respondents mentioned using only one standard (*n* = 28, 78%), while some respondents utilized two or three standards (*n* = 9, 25%) PRESS was the most frequently mentioned search strategy standard used (*n* = 13, 36%, Additional file 1: Table S4).

The majority of librarians who had peer reviewed (*n* = 40, 95%) rejected or recommended revisions of a manuscript. The most frequent reason given for manuscript rejection or revision was the “search methodology” (*n* = 36, 86%), followed by “entire article” (*n* = 34, 81%), then “search write-up” (*n* = 29, 69%). Respondents also listed “other reasons” (*n* = 23, 55%) for their decisions: the PRISMA flow diagram; tables of included, excluded, and ongoing studies; data extraction; inconsistent/incomplete reporting; pooling methods; and failure to use risk of bias tools. Note that these reasons could be identified as elements of the search methodology and of the search write-up, but respondents did not classify them in this way (Q8).

When asked if they had declined a request from journal editors to peer review SR manuscripts, almost half (*n* = 20, 45%) reported they had declined (Q10). Top reasons included “not enough time” (*n* = 12, 60%) and “did not have enough expertise” (*n* = 10, 50%). One respondent said “I was asked to review the entire SR, which I did not feel competent to do. Had they asked for the search methods / search strategy only, I would have been happy to do so” (Q11).

Further analysis of the data showed that professional setting, if reported, made little difference in whether or not respondents had been asked by journal editors to peer review. Among librarians who were invited to peer review, the majority (*n* = 39, 62%) had participated in SR projects themselves at least 5 times. Fisher’s exact test shows that previous systematic review authorship is indeed associated with invitations to peer review SR submissions (Table 1).

**Table 1:** Association of librarians’ workplace and authorship experience with invitations to peer review systematic reviews.

Characteristic	No. of librarians who were invited to peer review	No. of librarians who were not invited to peer review	P*
Workplace			0.62
Academic medical library (*n* = 169)	28	141	
Non-teaching hospital library (*n* = 4)	1	3	
Other (*n* = 48)	9	39	
Teaching hospital (*n* = 37)	4	33	
No. of published papers			
0 (*n* = 24)	1	23	< 0.001
1–4 (*n* = 79)	2	77	
5–10 (*n* = 61)	9	52	
11+ (*n* = 95)	30	65	

*Fisher test (does not include “no response”)

## Discussion

Our study has shown that the majority of librarians surveyed (*n* = 228, 78%) have not been invited to peer review systematic review manuscripts and that half (*n* = 122, 54%) of those not yet asked would be willing to do so. We also know that many editors struggle to find qualified peer reviewers [40]. This suggests that journal editors need ways to identify librarians who are interested in and capable of peer reviewing the search strategies and/or overall methodologies of manuscripts. Potentially a registry of qualified librarians could be developed and made available to editors. To help editors find a good match, the registry could include librarians’ experience with systematic reviews, and their areas of expertise. Journal editors could also look at SR search methods papers to identify qualified search specialists. Some automated tools help identify appropriate reviewers, such as Jane (Journal/Author Name Estimator) and PubReMiner [41, 42]. Librarians who wish to peer review SRs should also explore existing peer reviewer registries, such as Publons, and make their profiles available on multiple platforms to increase their professional visibility and help journal editors find them [43]. Librarians who are already registered in journal submission systems as an author or those who proactively choose to register could indicate that they wish to peer review—often by simply checking “yes” during registration. However, many current submission systems do not capture librarians’ areas of expertise and skills in their pre-defined list of keywords or classifications or require that a minimum number of terms be selected, leaving librarians forced to choose from medical specialties or vague terms like “education” or “administration.” Some systems, like Editorial Manager or ScholarOne Manuscripts, allow journals to enable personal keywords beyond the pre-defined lists, but many journals have not enabled this option. There is an opportunity for advocacy with journal editors and software manufacturers to expand registration and profile options and establish some pre-defined options in journal submission systems (like “information specialist/librarian” or “systematic reviews” or “search specialist”). Promoting new ways to match qualified librarians with editors could help improve the peer review of systematic review manuscripts.

In our survey, 32% of information specialists/librarians (*n* = 20) declined invitations to peer review entire manuscripts and only half of those who have not yet been asked (*n* = 122, 54%) expressed willingness to peer review. Even though many librarians are expert systematic review methodologists and searchers, they may lack skills in peer reviewing and knowledge of the scientific content [10, 44]. In addition to time limitations that all peer reviewers face, librarians might be reluctant to volunteer due to a perceived lack of expertise in peer review. It is important to acknowledge that there are different levels of expertise in the information specialist/librarian community and that the variation in breadth and depth of this expertise is likely reflected in our findings. With increased training and clear guidelines about which sections they are being asked to review, librarians might be more likely to accept invitations to peer review, adding to the pool of potential reviewers and improving published SRs.

One way of doing this would be for editors to ask librarians to review only specific sections of manuscripts, such as the methodology and search strategies, to harness their specialized expertise. Librarians who would like to gain peer review fluency could seek to increase their skills with the PRESS tool and through online peer reviewer training [31, 45]. Professional organizations, library associations, and journal editors could also offer specific peer review training to librarians and maintain a searchable bank of librarian peer reviewers. Library associations and other stakeholders, like the International Committee of Medical Journal Editors (ICMJE), could advocate to journal editors that librarian peer reviewers could improve search and methodological quality, reporting, and reproducibility [46].

The most prominent standards and guides recommend librarians be involved in systematics reviews. The Campbell Collaboration “requires the expertise . . . of an information specialist (IS) or a librarian” for information retrieval because it is a crucial part of the systematic review process [9]. The 2019 draft of the sixth edition of the Cochrane Handbook for Systematic Reviews of Interventions defines an integral role for the information specialist/librarian in the production of systematic reviews and recommends that authors work closely, from the start of the protocol, with a librarian experienced in the process [47]. The National Academies of Sciences, Engineering and Medicine recommend that teams work with a librarian to plan and peer review the search strategy [4]. Yet most biomedical editorial policies do not require librarian peer review of search methodologies submitted in manuscripts. Some journals, such as *Ophthalmology*, *Academic Medicine*, *Journal of School Nursing*, and *Annals of Family Medicine* and those listed in Additional file 1: Table S2 have turned to librarians and information specialists for peer review. Editors from other journals may not be aware that librarians have this expertise and are willing to take on this role. Biostatisticians have increasingly made the case that a biostatistician should review manuscripts’ statistical analyses [48, 49]. Journal editors could adopt this model for librarian peer review of systematic review searches and methods.

Another important but perhaps not unexpected finding is that librarians were more likely to be asked to peer review a manuscript if they had a record of systematic review co-authorship. Co-authoring a published SR or serving on a systematic review team as a methodologist and expert searcher can demonstrate a level of expertise necessary for peer reviewing manuscripts. Editors who seek peer reviewers can discover some librarians more easily, perhaps from their record of publications, long service, and existing registrations on journal submission systems. Two thirds of respondents (*n* = 194, 67%) had participated in SR teams but had not been asked to peer review a journal manuscript (Table 1). This group reported participating in at least one systematic review and up to 40, with more than half having participated in at least five systematic review teams (Additional file : Table S1—Q14). There is clearly a pool of untapped experts potentially available for peer review.

Librarians regularly refer to standards when designing, deploying, and reporting search strategies and methodologies for systematic reviews. PRISMA is a well-known standard for SR reporting elements and PRESS is a guideline for peer reviewing search strategies. Our survey revealed that very few respondents use both of these tools to review SRs. Respondents referred to PRISMA for reviewing the methods, but some librarians also reported using PRISMA to review the search strategy. This may reflect a lack of awareness of PRESS or an overreliance on PRISMA to simply assess the reporting of search methodologies, as opposed to the underlying quality and intellectual rigor of the search strategies themselves. As librarians gain more experience with the systematic review process, whether through years of experience, training, or involvement on SR projects, there may be less reliance on checklists and tools and more reliance on professional judgment. Librarians—and all reviewers—should refer to standards, checklists, and tools when peer reviewing [34, 50, 51]. The increased use of standards could improve the reliability and validity of peer review and, most importantly, the rigor of published systematic reviews. In fact, studies show that adherence to reporting guidelines and including a methodologist in peer review can lead to more citations [52] although that does not necessarily reflect the quality of the underlying search. Interestingly, since this survey was administered, a new PRISMA standard, PRISMA-S, has been released to serve as a reporting standard for searches to improve their transparency and reproducibility [11].

The reproducibility of the search in a systematic review or meta-analysis is one of the markers of a high-quality review [29]. As experts in literature searching as well as systematic review methodology, information specialists and librarians are able to critically assess the quality of search strategies and reporting. This study revealed that very few librarians who peer reviewed a manuscript found the search or the reporting methods of fully acceptable quality and rigor. The majority of respondents (*n* = 40, 95%) rejected or recommended revisions to manuscripts they peer reviewed, reflecting the overall publication process where very few papers are accepted outright in journals [53]. Librarians who serve as peer reviewers for journals are not simply rubber stamping the manuscripts that they review. They bring their experience, knowledge of established tools and standards, as well as their professional judgment to this role [35]. The addition of a librarian with searching expertise and methodological experience to the peer review process for submitted systematic reviews should improve the integrity of the search strategies and methods and thus the data underlying the entire review, which should, in turn, improve the quality of published systematic reviews to inform health care decision-making.

## Limitations

This study had several limitations. We used a non-validated survey instrument for this novel project. Its measurement properties, as described by the COSMIN definitions, are unknown, including its reliability, validity, responsiveness, and interpretability [54]. We are unaware of any validated survey instruments designed to measure the experiences of peer reviewers in general, let alone librarian peer reviewers. Survey results are based on self-reported responses, and the survey is likely to have attracted a non-representative sample of respondents with peer reviewing experience. It might have attracted librarians and information specialists who have more experience with systematic reviews, even though respondents with no experience were also encouraged to complete the survey. Respondents were asked to recall events in the past. For example, we asked respondents to estimate the number of manuscripts they had peer reviewed. Because of the use of professional biomedical librarian listservs to recruit respondents, we are unable to report a response rate, nor do we know how representative the participants are. Despite pilot-tested language in the survey, some responses clearly are referring to informal pre-submission peer review of searches by librarian colleagues rather than journal-level formal peer review of manuscripts. The terms “systematic review” and “meta-analysis” were not clearly defined and may have been interpreted inconsistently by respondents. This study did not explore whether a single reviewer is adequate to peer review the search strategy of a systematic review; further studies could examine inter-rater reliability of librarians as peer reviewers. All authors are or were practicing biomedical librarians, which could have introduced bias to the survey or manuscript.

## Conclusion

This survey reports medical librarian and information specialists’ experience peer reviewing systematic review manuscripts submitted for publication. Librarians are highly qualified to do comprehensive searching and often participate in systematic review teams. Furthermore, literature has shown that librarian involvement in production of a systematic review increases its quality. However, only a quarter of librarians in our study were involved in peer review of systematic reviews. Those who were involved were tough reviewers and overwhelmingly rejected or recommended revisions to manuscripts. More effort is needed from publishers, editors, journals, and professional library associations to increase the rates of librarian, information specialist, or other search strategy experts’ involvement in evaluation of systematic review manuscripts.

## Supplementary information

**Additional file 1:** Survey Questions. **Table S1.** Survey Results (Q2-Q16). **Table S2.** Complete List of Journal Titles for Which Survey Respondents Have Peer Reviewed a Systematic Review or Meta-Analysis Manuscript (Q3). **Table S3.** Standards Used to Evaluate the Methods Section of the Manuscript (Q6). **Table S4.** Standards Used to Evaluate Search Strategies (Q7). **Table S5.** Survey Questions. **Table S6.** CHERRIES-Compliant Reporting Checklist.

## Data Availability

Data and materials are available on the Open Science Framework at https://osf.io/s6yab/.

## Abbreviations

AASHL: Association of Academic Health Sciences Libraries
CHERRIES: Checklist for Reporting Results of Internet E-Surveys
COSMIN: Consensus-Based Standards for the Selection of Health Measurement Instruments
EQUATOR: Enhancing the Quality and Transparency of Health Research
IOM: Institute of Medicine
JBI: Joanna Briggs Institute
MA: Meta-Analysis
MECIR: Methodological Expectations of Cochrane Intervention Reviews
MOOSE: Meta-analyses Of Observational Studies in Epidemiology
PRESS: Peer Review of Electronic Search Strategies
PRISMA: Preferred Reporting Items for Systematic Reviews and Meta-Analyses
PRISMA-S: PRISMA Search Reporting Extension
SR: Systematic Review
STROBE: Strengthening the Reporting of Observational Studies in Epidemiology
