# Supplementary material for: Librarians as methodological peer reviewers for systematic reviews: results of an online survey
Source: Res Integr Peer Rev. 2019 Nov 27;4:23. doi: 10.1186/s41073-019-0083-5 (PMC6882225; doi:10.1186/s41073-019-0083-5)
Supplement: Supplementary file 1 — Additional file 1: Survey Questions. Table S1. Survey Results (Q2-Q16). Table S2. Complete List of Journal Titles for Which Survey Respondents Have Peer Reviewed a Systematic Review or Meta-Analysis Manuscript (Q3). Table S3. Standards Used to Evaluate the Methods Section of the Manuscript (Q6). Table S4. Standards Used to Evaluate Search Strategies (Q7). Table S5. Survey Questions. Table S6. CHERRIES-Compliant Reporting Checklist. [file 41073_2019_83_MOESM1_ESM.docx]

**Additional File - Librarians as methodological peer reviewers for systematic reviews: results of an online survey**

Holly K. Grossetta Nardini, Janene Batten, Melissa C. Funaro, Rolando Garcia-Milian, Kate Nyhan, Judy M. Spak, Lei Wang, Janis G. Glover

Harvey Cushing/John Hay Whitney Medical Library, Yale University, 333 Cedar Street, New Haven, CT 06520-8014

Corresponding author: holly.nardini@yale.edu

**Table S1**

**Survey Results**

| **Q2.** Has a journal editor asked you to peer review a systematic review or meta-analysis manuscript that has been submitted to the journal for publication? | | | |
| --- | --- | --- | --- |
| All respondents (n=**291**) saw this question.  There are **291** responses (100%) to this question. | | | |
|  | | | |
| **Responses** | **Count** | **% of total (n=291)** | **% of responses (n=291)** |
| No | 228 | 78.35% | 78.35% |
| Yes | 63 | 21.65% | 21.65% |

| **Q3.** Which journal(s)? | | | |
| --- | --- | --- | --- |
| Only respondents who answered “Yes” in Q2 (**n=63**) saw this question.  There are **40** responses (63.49%) to this question. Multiple answers per response allowed.  The most frequently mentioned journals are listed below. See more in Table S2. | | | |
|  | | | |
| **Journals** | **Count** | **% of total (n=63)** | **% of responses (n=40)** |
| PLoS one | 5 | 7.94% | 12.50% |
| JAMIA: a scholarly journal of informatics in health and biomedicine | 3 | 4.76% | 7.50% |
| JBI database of systematic reviews | 3 | 4.76% | 7.50% |
| Journal of the Medical Library Association | 3 | 4.76% | 7.50% |
| Systematic reviews | 3 | 4.76% | 7.50% |
| Annals of internal medicine | 2 | 3.17% | 5.00% |
| Cochrane database of systematic reviews | 2 | 3.17% | 5.00% |
| Research synthesis methods | 2 | 3.17% | 5.00% |

| **Q4.** Do you know why you were asked (e.g. professional expertise, standards required it)? | | | |
| --- | --- | --- | --- |
| Only respondents who answered “Yes” in Q2 (**n=63**) saw this question.  There are **44** responses (69.84%) to this question. | | | |
|  | | | |
| **Responses** | **Count** | **% of total (n=63)** | **% of responses (n=44)** |
| Yes | 31 | 49.21% | 70.45% |
| No | 13 | 20.63% | 29.55% |
| No Response | 19 | 30.16% | N/A |

**Q5.** How many systematic review or meta-analysis manuscripts have you peer reviewed?

Only respondents who answered “Yes” in Q2 (**n=63**) saw this question.

There are **43** responses (68.25%) to this question.

Mean: 5.62791

Median: 4

Standard Deviation: 7.480206

Skewness: 3.022307

Kurtosis: 10.751138

The value of the K-S test statistic (D) is .32454.

The p-value is .00015. Data not normally distributed.

Lower quartile (x_L_): 1

Upper quartile (x_U_): 5

Interquartile range (x_U_-x_L_): 4

| **Q6.** What standards, if any, did you use to evaluate the methods section of the manuscript? | | | |
| --- | --- | --- | --- |
| Only respondents who answered “Yes” in Q2 (**n=63**) saw this question.  There are **37** responses (58.73%) to this question. Multiple answers per response allowed.  The most frequently mentioned standards are listed below. See more in Table S3. | | | |
|  | | | |
| **Standards** | **Count** | **% of total (n=63)** | **% of responses (n=37)** |
| PRISMA or PRISMA-P (Preferred Reporting Items for Systematic review and Meta-Analysis Protocols) and other extensions | 32 | 50.79% | 86.49% |
| *Cochrane Handbook* or Cochrane | 10 | 15.87% | 27.03% |
| Professional judgement | 8 | 12.70% | 21.62% |
| MECIR (Methodological Expectations of Cochrane Intervention Reviews) | 4 | 6.35% | 10.81% |
| Journal expectations | 3 | 4.76% | 8.11% |
| IOM (Institute of Medicine) | 2 | 3.17% | 5.41% |
| CASP (Critical Appraisal Skills Programme) | 2 | 3.17% | 5.41% |
| STROBE (Strengthening the Reporting of Observational Studies in Epidemiology) | 2 | 3.17% | 5.41% |

| **Q7.** What standards, if any, did you use to evaluate the search strategy? | | | |
| --- | --- | --- | --- |
| Only respondents who answered “Yes” in Q2 (**n=63**) saw this question.  There are **36** responses (57.14%) to this question. Multiple answers per response allowed. | | | |
|  | | | |
| **Standards** | **Count** | **% of total (n=63)** | **% of responses (n=36)** |
| PRESS (Peer Review of Electronic Search Strategies) | 13 | 20.63% | 36.11% |
| Professional judgement | 11 | 17.46% | 30.56% |
| PRISMA (Preferred Reporting Items for Systematic Reviews and Meta-Analyses) | 9 | 14.29% | 25.00% |
| *Cochrane Handbook* or Cochrane | 8 | 12.70% | 22.22% |
| MECIR (Methodological Expectations of Cochrane Intervention Reviews) | 3 | 4.76% | 8.33% |
| Journal expectations | 3 | 4.76% | 8.33% |
| IOM (Institute of Medicine) | 2 | 3.17% | 5.56% |
| JBI (Joanna Briggs Institute) | 1 | 1.59% | 2.78% |

| **Q8.** Have you ever rejected or recommended a revision of a manuscript based on the following? | | | |
| --- | --- | --- | --- |
| Only respondents who answered “Yes” in Q2 (**n=63**) saw this question.  There are **42** responses (66.67%) to this question. Multiple answers per response allowed. | | | |
|  | | | |
| **Reasons for Rejection or Revision** | **Count** | **% of total (n=63)** | **% of responses (n=42)** |
| Search Methodology | 36 | 57.14% | 85.71% |
| Entire Article | 34 | 53.97% | 80.95% |
| Search Write-Up | 29 | 46.03% | 69.05% |
| Other | 23 | 36.51% | 54.76% |
| Never Rejected | 2 | 3.17% | 4.76% |

| **Q9.** Would you be willing to discuss your involvement with peer reviewing systematic review or meta-analysis manuscripts for publication? | | | |
| --- | --- | --- | --- |
| Only respondents who answered “Yes” in Q2 (**n=63**) saw this question.  There are **41** responses (65.08%) to this question. | | | |
|  | | | |
| **Responses** | **Count** | **% of total (n=63)** | **% of responses (n=41)** |
| Yes | 27 | 42.86% | 65.85% |
| No | 14 | 22.22% | 34.15% |
| No Response | 12 | 34.92% | N/A |

| **Q10.** Have you ever declined a request from a journal editor to peer review a systematic review or meta-analysis manuscript? | | | |
| --- | --- | --- | --- |
| Only respondents who answered “Yes” in Q2 (**n=63**) saw this question.  There are **44** responses (69.84%) to this question. | | | |
|  | | | |
| **Responses** | **Count** | **% of total (n=63)** | **% of responses (n=44)** |
| Yes | 20 | 31.75% | 45.45% |
| No | 24 | 38.10% | 54.55% |
| No Response | 19 | 30.16% | N/A |

| **Q11.** Why did you decline the request from the journal editor to peer review the systematic review or meta-analysis manuscript? | | | |
| --- | --- | --- | --- |
| Only respondents who answered “Yes” in Q10 (**n=20**) saw this question.  There are **20** responses (100%) to this question. Multiple answers per response allowed.  The most frequent reasons are listed below. | | | |
|  | | | |
| **Reasons for Declining Requests** | **Count** | **% of total (n=20)** | **% of responses (n=20)** |
| Not Enough Time | 12 | 60.00% | 60.00% |
| Did Not Have Enough Expertise | 10 | 50.00% | 50.00% |

| **Q12.** What kind of library do you work in? | | | | |
| --- | --- | --- | --- | --- |
| All respondents (**n=291**) saw this question.  There are **258** responses (88.66%) to this question. | | | | |
|  | | | | |
| **Responses** | | **Count** | **% of total (n=291)** | **% of responses (n=258)** |
| Academic Medical Library | | 169 | 58.08% | 65.50% |
| Teaching Hospital Library | | 37 | 12.71% | 14.34% |
| Non-Teaching Hospital Library | | 4 | 1.37% | 1.55% |
| Other | | 48 | 16.49% | 18.60% |
| Research Center | 9 |  |  |  |
| Academic Library | 9 |  |  |  |
| Association/Organization | 9 |  |  |  |
| Government | 5 |  |  |  |
| Other Health Care Institution | 5 |  |  |  |
| Both Academic Medical Library and Teaching Hospital | 4 |  |  |  |
| Other Library | 2 |  |  |  |
| Other Educational Institution | 1 |  |  |  |
| Not specified | 4 |  |  |  |
| No Response | | 33 | 11.34% | N/A |

**Q13.** How many years have you been a librarian?

All respondents (**n=291**) saw this question.

There are **256** responses (87.97%) to this question.

Mean: 14.10547

Median: 11.5

Standard Deviation: 10.368249

Skewness: 0.850936

Kurtosis: -0.012967

The value of the K-S test statistic (D) is .12688.

The p-value is .00047. Data not normally distributed.

Lower quartile (x_L_): 6

Upper quartile (x_U_): 20

Interquartile range (x_U_-x_L_): 14

| **Q14.** How many times have you participated on a systematic review team, or created and conducted searches for systematic reviews, etc.? | | | |
| --- | --- | --- | --- |
| All respondents (**n=291**) saw this question.  There are **259** responses (89.00%) to this question. | | | |
|  | | | |
| **Responses** | **Count** | **% of total (n=291)** | **% of responses (n=259)** |
| 11+ | 95 | 32.65% | 36.68% |
| 5-10 | 61 | 20.96% | 23.55% |
| 1-4 | 79 | 27.15% | 30.50% |
| 0 | 24 | 8.25% | 9.27% |
| No Response | 32 | 11.00% | N/A |

| **Q15.** How have you trained to participate in conducting systematic reviews? | | | |
| --- | --- | --- | --- |
| All respondents (**n=291**) saw this question.  There are **259** responses (89.00%) to this question. Multiple answers per response allowed. | | | |
|  | | | |
| **Training Types** | **Count** | **% of total (n=291)** | **% of responses (n=259)** |
| Attended classes/webinars | 220 | 75.60% | 84.94% |
| In-house training | 121 | 41.58% | 46.72% |
| Self-trained | 145 | 49.83% | 55.98% |
| Other | 29 | 9.97% | 11.20% |
| No training | 12 | 4.12% | 4.63% |

| **Q16.** If given the opportunity, would you serve as a peer reviewer for a systematic review or meta-analysis manuscript that has been submitted to a journal for publication? | | | |
| --- | --- | --- | --- |
| Only respondents who answered “No” in Q2 (**n=228**) saw this question.  There are **224** responses (98.25%) to this question. | | | |
|  | | | |
| **Responses** | **Count** | **% of total (n=228)** | **% of responses (n=224)** |
| Yes | 122 | 53.51% | 54.46% |
| Maybe | 93 | 40.79% | 41.52% |
| No | 9 | 3.95% | 4.02% |
| No Response | 4 | 1.75% | N/A |

**Table S2**

**Complete List of Journal Titles for Which Survey Respondents Have Peer Reviewed a Systematic Review or Meta-Analysis Manuscript (Q3)**

| **Journal Title** | **Number** |
| --- | --- |
| PLOS ONE | 5 |
| JAMIA: a scholarly journal of informatics in health and biomedicine | 3 |
| JBI database of systematic reviews | 3 |
| Journal of the Medical Library Association | 3 |
| Systematic reviews | 3 |
| Annals of internal medicine | 2 |
| Cochrane database of systematic reviews | 2 |
| Research synthesis methods | 2 |
| American journal of respiratory and critical care medicine | 1 |
| Animal: An international journal of animal bioscience | 1 |
| Annals of emergency medicine | 1 |
| Applied clinical informatics | 1 |
| Archives of physical medicine and rehabilitation | 1 |
| BMC systematic reviews | 1 |
| BMJ open | 1 |
| Canadian journal of pain | 1 |
| Clinical and experimental dermatology | 1 |
| Clinical and investigative medicine, the journal of the Canadian Society of Clinical Investigation | 1 |
| Clinical rehabilitation | 1 |
| Clinical trials journal of the Society for Clinical Trials | 1 |
| EJEL: the electronic journal of e-learning | 1 |
| European respiratory journal | 1 |
| Evidence based library and information practice | 1 |
| Gerontechnology | 1 |
| Health information & libraries journal | 1 |
| International journal of nursing | 1 |
| Journal of behavioral health services and research | 1 |
| Journal of clinical epidemiology | 1 |
| Journal of communication in healthcare: strategies, media and engagement in global health | 1 |
| Journal of medical Internet research | 1 |
| Journal of the academy of nutrition and dietetics | 1 |
| Journal of patient-reported outcomes | 1 |
| Journal of school nurses | 1 |
| NEJM | 1 |
| Nurse educator | 1 |
| Ophthalmology | 1 |
| Zoonoses and public health | 1 |

**Table S3**

**Standards Used to Evaluate the Methods Section of the Manuscript (Q6)**

|  | **Number of Times Reported** |
| --- | --- |
| PRISMA or PRISMA-P (Preferred Reporting Items for Systematic review and Meta-Analysis Protocols) and other extensions | 32 |
| *Cochrane Handbook* or Cochrane | 10 |
| Professional judgement | 8 |
| MECIR (Methodological Expectations of Cochrane Intervention Reviews) | 4 |
| Journal expectations | 3 |
| IOM (Institute of Medicine) | 2 |
| CASP (Critical Appraisal Skills Programme) | 2 |
| STROBE (Strengthening the Reporting of Observational Studies in Epidemiology) | 2 |
| MOOSE (Meta-Analyses Of Observational Studies in Epidemiology) | 1 |
| AHRQ (Agency for Healthcare Research and Quality) | 1 |
| STARD (Standard for Reporting Diagnostic Accuracy) | 1 |
| CONSORT (Consolidated Standards of Reporting Trials) | 1 |
| PRESS (Peer Review of Electronic Search Strategies) | 1 |

**Table S4**

**Standards Used to Evaluate Search Strategies (Q7)**

|  | **Number of Times Reported** |
| --- | --- |
| PRESS (Peer Review of Electronic Search Strategies) | 13 |
| Professional judgement | 11 |
| PRISMA (Preferred Reporting Items for Systematic Reviews and Meta-Analyses) | 9 |
| *Cochrane Handbook* or Cochrane | 8 |
| MECIR (Methodological Expectations of Cochrane Intervention Reviews) | 3 |
| Journal expectations | 3 |
| IOM (Institute of Medicine) | 2 |
| JBI (Joanna Briggs Institute) | 1 |

**Table S5**

**Survey Questions**

Q1

Thank you for your interest in participating in the research study being conducted by the librarians at the Cushing/Whitney Medical Library at Yale University. The results of this study will be reported at the Medical Library Association’s Annual Meeting in Atlanta in May 2018.

**Explanation of the Research and What You Will Do**

This study investigates librarian involvement in reviewing systematic reviews, specifically how frequently librarians are serving as journal reviewers for the methodology of systematic review manuscripts. You will be asked a series of questions about how frequently you are involved in peer reviewing systematic reviews as part of the journal peer review process. The survey will take approximately 5 minutes.

**Content of the Survey**

In addition to survey questions regarding your involvement as a librarian peer reviewer, we will ask questions about your type of library and what systematic review training that you might have had. We ask if you would be willing to discuss your involvement with peer reviewing and provide a box for your contact information. Personal names will not be published or referenced. Comments will be reported anonymously or in aggregation.

**Your Right to Participate, Say No, or Withdraw**

Participation in this research project is completely voluntary. You have the right to say no. You may change your mind at any time and withdraw. You may choose not to answer specific questions or to stop participating at any time.

**Costs and Compensation for Being in the Study**

There are no costs to you for participating and you will not be compensated.

**Benefits and Risks**

There are no personal benefits or risks to participating in this study.

**Contact Information for Questions and Concerns**

The Institutional Review Board (IRB) at Yale University has reviewed this study and deemed it exempt. If you have concerns or questions about this study, please contact the researcher: Holly Grossetta Nardini, Associate Director, Cushing/Whitney Medical Library, Yale University. [holly.nardini@yale.edu]

Clicking the AGREE button below indicates that I have read the previous information and agree to volunteer to participate in this survey.

Q2. Has a journal editor asked you to peer review a systematic review or meta-analysis manuscript that has been submitted to the journal for publication?

Q3. Which journal(s)?

Q4. Do you know why you were asked (e.g. professional expertise, standards required it)?

Q5. How many systematic review or meta-analysis manuscripts have you peer reviewed? (Estimates are fine)

Q6. What standards, if any, did you use to evaluate the methods section of the manuscript?

Q7. What standards, if any, did you use to evaluate the search strategy?

Q8. Have you ever rejected or recommended a revision of a manuscript based on the following:

Search write-up

Search methodology

Entire article

Other

No, I have never rejected or recommended a revision

Q9. Would you be willing to discuss your involvement with peer reviewing systematic review or meta-analysis manuscripts for publication?

Q10. Have you ever declined a request from a journal editor to peer review a systematic review or meta-analysis manuscript?

Q11. Why did you decline the request from the journal editor to peer review the systematic review or meta-analysis manuscript?

Q12. What kind of library do you work in?

Q13. How many years have you been a librarian?

Q14. How many times have you participated on a systematic review team, or created and conducted searches for systematic reviews, etc.?

Q15. How have you trained to participate in conducting systematic reviews?

Q16. If given the opportunity, would you serve as a peer reviewer for a systematic review or meta-analysis manuscript that has been submitted to a journal for publication?

**Table S6**

**CHERRIES-Compliant Reporting Checklist**

| Item Category | Checklist Item | Response |
| --- | --- | --- |
| Design | describe survey design | We designed this survey for a purposive sample of medical librarians. |
| IRB approval and informed consent process | IRB approval | The Institutional Review Board at Yale University reviewed this study and deemed it exempt from human subjects protection (IRB #2000022848). |
|  | informed consent | We placed informed consent language approved by the IRB at the beginning of the survey. The informed consent language told participants the length of time of the survey (approximately five minutes), who the primary investigator was (Holly Grossetta Nardini), and the purpose of the study (to investigate librarian involvement in reviewing systematic reviews). The informed consent language did not tell participants which data were stored and where and for how long. |
|  | data protection | We collected limited personal information: participants who were willing to discuss librarian involvement in reviewing systematic reviews were invited to supply their email addresses. We stored this personal information in Qualtrics, a HIPAA-compliant survey tool. We did not include this personal information in the data uploaded to the Open Science Framework. |
| development and pre-testing | development and testing | A methodological expert with knowledge of user experience and the Qualtrics platform reviewed the survey, and we pilot tested the survey with a group of medical librarians. |
| recruitment process and description of the sample having access to the questionnaire | open survey versus closed survey | This survey was an open survey. |
|  | contact mode | The initial contact with the potential participants was made by email. |
|  | advertising the survey | We distributed the survey through three listservs for information professionals in medical libraries: AAHSL-all (approximately 350 members in June 2019), medlib-l (approximately 2100 members in June 2019), and expertsearcher (approximately 1500 members in June 2019). There is an unknown degree of overlap in the membership of these listservs. Recipients were invited to share the survey link beyond the three listservs. |
| survey administration | web/email | This survey was a web survey hosted on Qualtrics. |
|  | context | The three listservs were the survey was distributed all cater to medical librarians. The Association of Academic Health Sciences Libraries runs AAHSL-all; the Medical Library Association owns MEDLIB-L and expertsearching. |
|  | mandatory/voluntary | This survey was voluntary. |
|  | incentives | We did not offer incentives. |
|  | time/date | Data were collected between March 15, 2018 and April 6, 2018. |
|  | randomization of items or questionnaires | This survey had no randomization or alternation. |
|  | adaptive questioning | We used branching logic to conditionally display certain items (questions about peer reviewing experiences) based on answers to earlier questions. |
|  | number of items | This survey included 16 items (if a participant's responses led to the display of all branches). |
|  | number of screens (pages) | This survey included 4 screens (if a participant's responses led to the display of all branches). |
|  | completeness check | This survey did not include any completeness check. |
|  | review step | This survey did not present participants with a summary of the responses. However, participants could use a "previous page" button to return to earlier pages and select different answers. |
| response rates | unique site visitor | While our survey platform, Qualtrics, does record respondents' IP addresses, we believe that multiple medical librarians working at the same institution might have responded to the survey from the same IP address, and so we have not attempted to determine unique visitors. |
|  | view rate | The view rate cannot be calculated without a unique site visitor number. |
|  | participation rate | The participation rate cannot be calculated without a unique site visitor number. |
|  | completion rate | 266 participants submitted at least one response on the last survey page shown to them. 302 participants started the survey. Thus the completion rate was 88%. |
| preventing multiple entries from the same individual | cookies used | We did not use cookies to assign unique user identifiers, and so it is possible that some participants responded to this survey more than once. |
|  | IP check | We did not use participants' IP addresses to limit the number of entries, and so it is possible that some participants responded to this survey more than once. |
|  | log file analysis | We investigated log files and found at least one clear-cut example of three participants from the same IP address starting the survey around the same time (immediately after we sent the survey announcement to listservs). The three participants finished the survey at different times and gave different responses, so we believe that they were indeed three unique individuals who happen to share the same IP address at a workplace. We did not exclude any responses, even partial ones, based on log file analysis. |
|  | registration | This survey was open, so this item does not apply. |
| analysis | handling of incomplete questionnaires | We analyzed partially completed questionnaires. |
|  | questionnaires submitted with an atypical timestamp | We did not impose a cut-off time. |
|  | statistical correction | We did not adjust for the non-representative sample. |
